# Long-Term Intrahost Evolution of *Staphylococcus aureus* Among Diabetic Patients With Foot Infections

**DOI:** 10.3389/fmicb.2021.741406

**Published:** 2021-09-06

**Authors:** Jean-Philippe Lavigne, Michel Hosny, Catherine Dunyach-Remy, Adeline Boutet-Dubois, Sophie Schuldiner, Nicolas Cellier, Alex Yahiaoui-Martinez, Virginie Molle, Bernard La Scola, Hélène Marchandin, Albert Sotto

**Affiliations:** ^1^VBIC, INSERM U1047, Service de Microbiologie et Hygiène Hospitalière, Université de Montpellier, CHU Nîmes, Nîmes, France; ^2^Aix-Marseille Université UM63, Institut de Recherche pour le Développement IRD 198, Assistance Publique – Hôpitaux de Marseille (AP-HM), Microbes, Evolution, Phylogeny and Infection (MEΦI), Institut Hospitalo-Universitaire (IHU) – Méditerranée Infection, Marseille, France; ^3^VBIC, INSERM U1047, Service des Maladies Métaboliques et Endocriniennes, Université de Montpellier, CHU Nîmes, Nîmes, France; ^4^Service d’Orthopédie, CHU Nîmes, Nîmes, France; ^5^Laboratory of Pathogen Host Interactions, UMR 5235, CNRS, Université de Montpellier, Montpellier, France; ^6^HydroSciences Montpellier, CNRS, IRD, Service de Microbiologie et Hygiène Hospitalière, Université de Montpellier, CHU Nîmes, Nîmes, France; ^7^VBIC, INSERM U1047, Service des Maladies Infectieuses et Tropicales, Université de Montpellier, CHU Nîmes, Nîmes, France

**Keywords:** diabetic foot infection, genome adaptation, longitudinal evolution, methicillin-resistant *Staphylococcus aureus*, *Staphylococcus aureus*

## Abstract

*Staphylococcus aureus* is one of the main pathogens isolated from diabetic foot infections (DFI). The purpose of this study was to evaluate the importance of the persistence of *S. aureus* in this environment and the possible modifications of the bacterial genome content over time. Molecular typing of *S. aureus* isolates cultured from patients with the same DFI over a 7-year study revealed a 25% rate of persistence of this species in 48 patients, with a short median persistence time of 12weeks (range: 4–52weeks). Non-specific clonal complexes were linked to this persistence. During the follow-up, *bla* genes were acquired in three cases, whereas some virulence markers were lost in all cases after a long period of colonization (21.5weeks). Only one patient (2%) had a long-term persistence of 48weeks. The genome sequencing of a clonal pair of early/late strains isolated in this patient showed mutations in genes encoding bacterial defence and two-component signal transduction systems. Although, this study suggests that the long-term persistence of *S. aureus* in DFI is a rare event, genomic evolution is observed, highlighting the low adaptive ability of *S. aureus* to the specific environment and stressful conditions of diabetic foot ulcers. These results provide the basis for better understanding of *S. aureus* dynamics during persistent colonization in chronic wounds.

## Introduction

Chronic wounds correspond to those that do not progress through the healing process in an orderly and timely manner (Frykberg and Banks, 2015). The definition of time without complete or partial healing differs across countries, ranging from 4weeks to 3months (Järbrink et al., 2016). They include diabetic foot ulcers (DFU), venous leg ulcers, and pressure ulcers. Among them, DFU are one of the main complications in diabetic patients, with a lifetime prevalence varying between 15 and 25% (Armstrong et al., 2017). These wounds are frequently infected (more than 50%) and the infection that spreads to underlying soft tissues and bone structures is responsible for numerous lower-limb amputations (Senneville et al., 2020). Moreover, the consequences of DFU in terms of mortality, morbidity, and diabetes-related hospital admissions represent an important cost for society (Lipsky et al., 2020).

Several studies have shown that diabetic foot infections (DFI) are polymicrobial (Liu et al., 2020); however, *Staphylococcus aureus* is the most frequently isolated pathogen (Dunyach-Remy et al., 2016), mainly in Occidental countries. This species is found either alone or in combination, regardless of the depth of the infection. *Staphylococcus aureus* is both a commensal bacterium and an opportunistic human pathogen. Indeed, although approximately 30% of the human population is colonized with this species (Wertheim et al., 2005), it can also cause a wide range of clinical infections (e.g., bacteremia, endocarditis, skin and soft tissue, osteoarticular, pulmonary, and device-related infections; Tong et al., 2015). The numerous virulence factors and toxins produced by *S. aureus* are well characterized and responsible for the different clinical situations (Serra et al., 2015). One of the main problems in the management of DFI is the misuse of antibiotics. This is particularly frequent due to the mitigated clinical signs of ulcer infection. The unnecessary antimicrobial treatment has an impact on the bacterial microbiota and contributes to the selection and emergence of multidrug-resistant bacteria. Thus, the prevalence of methicillin-resistant *S. aureus* (MRSA) in DFI is high (Dunyach-Remy et al., 2016). Different risk factors for MRSA acquisition have been identified including prior hospitalization, living in a chronic care facility, previous antibiotic use, and previous amputation (Tentolouris et al., 2006; Ertugrul et al., 2012).

DFU colonization/infection by *S. aureus* is clearly described and *S. aureus* is known to hide and persist in skin cells and osteoblasts, thereby contributing to wound chronicity, yet very few data are available about the persistence rate of the species in this chronic infection. Longitudinal studies on persisting *S. aureus* strains in this context are lacking (Huitema et al., 2020). This contrasts with other chronic infections, the most well-known being lung infections in cystic fibrosis (*CF*) patients. In *CF*, longitudinal studies showed that *S. aureus* isolates belonging to the same genetic lineage are repeatedly recovered, indicating chronic infection with a single strain, and revealed adaptive modifications of the species during persistence under diverse biotic and abiotic selective pressures (Branger et al., 1996; Vu-Thien et al., 2010; McAdam et al., 2011).

This prompted us to investigate *S. aureus* persistence and adaptive ability during DFI. Indeed, during chronic DFU infection, *S. aureus* is also subjected to numerous selective pressures resulting from different antibiotic regimens, the host immune system, and the environment of the wound in uncontrolled diabetic patients and the presence of multiple microorganisms with colonizing and/or infecting potential, which interact with one another (Pouget et al., 2020). Here, we determined the rate and characteristics of *S. aureus* persistence in DFU based on the identification of the same lineage of *S. aureus* during a longitudinal study of DFI, and we performed a comparative genome analysis of sequential *S. aureus* strains isolated in the same patient to investigate the *in vivo* adaptative evolution.

## Materials and Methods

### Patient Samples

Patients included in this study belonged to three previous projects approved by the Sud Mediterranean III Ethical Committee [clinical trials no. NCT01212120 (period 2011–2012), NCT01551667 (period 2012–2016), and NCT02565940 (period 2015–2017)] and carried out in accordance with the Helsinki Declaration as revised in 2000. All patients gave written informed consent for participation in these studies. From 1st January 2011 to 31st December 2017, we prospectively enrolled all diabetic patients managed in the Diabetic Foot Clinic Gard Occitanie at the Nîmes University Hospital (France) with a suspected newly presenting episode of DFI, without systemic antibiotic treatment within the previous 15days and with at least one sample taken for bacterial culture. Every wound was assessed for presence and severity of infection by a trained diabetologist using the PEDIS classification of the IWGDF consensus conference (Lipsky et al., 2020). Some clinical data (antibiotic and antiseptics used) and the wound evolution (healing/non healing) were collected from the previous studies. After wound debridement, samples for bacterial culture were obtained by scraping and collecting debris by swabbing at the wound base, or by a tissue biopsy using the procedure previously described (Lipsky et al., 2020). All the samples were immediately sent to the Department of Microbiology.

### Bacterial Identification

*Staphylococcus aureus* identification was performed using either the VITEK® 2 GP ID card or the VITEK® MS system (bioMérieux, Marcy l’Etoile, France).

### Antimicrobial Susceptibility Testing

For each *S. aureus* isolate, antimicrobial susceptibility was determined by the disk diffusion method (BioRad, Marnes La Coquette, France) on Mueller-Hinton agar plates according to European Committee on Antimicrobial Susceptibility Testing (EUCAST) 2020.^1^ The minimum inhibitory concentration (MIC) of isolates to vancomycin and teicoplanin was determined by microbroth dilution (Umic®, Biocentric, Bandol, France) over a range of dilutions (0.25–4mg/L for vancomycin and 0.25–8mg/L for teicoplanin). A cefoxitin disk was used to screen isolates for methicillin resistance. To confirm the presence/absence of *mecA* and *mecC* genes, we performed specific PCR as previously described (Sahebnasagh et al., 2014).

### Oligonucleotide DNA Arrays Procedure

Microarray-based characterization was carried out on all the MRSA isolated during the study. The Alere StaphyType DNA microarray (Alere Technologies GmbH, Jena, Germany) was used according to protocols and procedures previously described (Monecke et al., 2008, 2011). The screened numerous markers simultaneously tested in 5h. The DNA microarray includes 333 *S*. *aureus* target sequences, including species markers, antimicrobial resistance, and virulence-associated genes, and SCC*mec*-associated genes. Primer and probe sequences have previously been published (Monecke et al., 2008, 2011). DNA was extracted from each *S. aureus* strain, and after amplification and hybridization, markers were identified. This assay determines the clonal complex (CC) of strains. The CC may be defined as a cluster of strains (clones) that are similar enough to be claimed to share a common origin. This group of strains is genetically related to a single ancestral clone. Raw data were interpreted as “positive,” “negative,” or “ambiguous” using a previously described algorithm (Monecke et al., 2008, 2011). The affiliation of isolates to the CC or Sequence Type (ST) as defined based on Multi Locus Sequence Typing and *spa*-typing was determined by an automated comparison of hybridization profiles to a collection of previously characterized reference strains (Monecke et al., 2008).

### Whole-Genome Analysis and Single-Nucleotide Polymorphism Identification Procedure

*Staphylococcus aureus* strains (*n*=2, strains C1BP152 and C01P19) isolated 48weeks apart from the same patient were sequenced. The strains were grown aerobically on 5% Columbia sheep blood agar plates (Becton Dickinson, United States) at 37°C for 24h. Genomic DNA was extracted by EZ1 DNA Tissue Kit (QIAGEN, Germany). Whole Genome Sequencing (WGS) was performed with an Illumina MiSeq sequencing system (Illumina, San Diego, CA, United States) using the paired-end (PE) read libraries (PE250) prepared by Nextera XT DNA Library Prep Kit (Illumina) following the manufacturer’s protocol. Raw reads were processed using FastQC (v.0.11.7) to assess data quality. The Cutadapter tool (v.1.16) implemented in Python (v.3.5.2) was used to remove residual PCR primers and filter low quality bases (Q_score<30) and short reads (<150bp). The filtered trimmed reads were included in the downstream analysis. Obtained reads were mapped against *S. aureus* NCTC 8325 genome (GenBank accession number: GCA_000013425.1), employing the CLC genomics workbench 7 (Qiagen Inc., Valencia, CA), using default parameters; length fraction: 0.5, similarity fraction: 0.8. The assembled contigs were processed by Prokka software for microbial genome annotation (Seemann, 2014). Virulence factor database (VFDB)^2^ was used to infer virulence factor-encoding genes from genome sequences (Chen et al., 2005). Antimicrobial resistance genes were obtained from ABRIcate with the ResFinder database on assembled genomes (Zankari et al., 2012; Bortolaia et al., 2020). The two WGS were aligned using the MAFFT software (Katoh and Standley, 2013). Single nucleotide polymorphism calls were made from the PE library raw reads. For SNP analysis, we employed the following software: SNP-sites for variants calling (Page et al., 2016) and SnpEff (v.1.3T) for SNP annotation in coding regions (Cingolani et al., 2012). SNP annotations of affected genes were searched within wild-type genome and their effects were classified according to mutation impact. Genes affected by stop gain mutations were searched in Uniprot database for virulence classification. Additional analyses were performed on WGS such as circular genome representation [BLAST Ring Image Generator software (BRIG); Alikhan et al., 2011].

### Statistical Analysis

Comparisons of the identification rate of the genotypes (either CC or ST) for the *S. aureus* isolated from DFI at inclusion and during follow-up and between the virulence genes content between clonal or non-clonally-related groups of *S. aureus* strains were performed using the Fisher’s exact test. Values of *p*<0.01 were considered as significant.

## Results

### Studied Population

**Figure 1:**
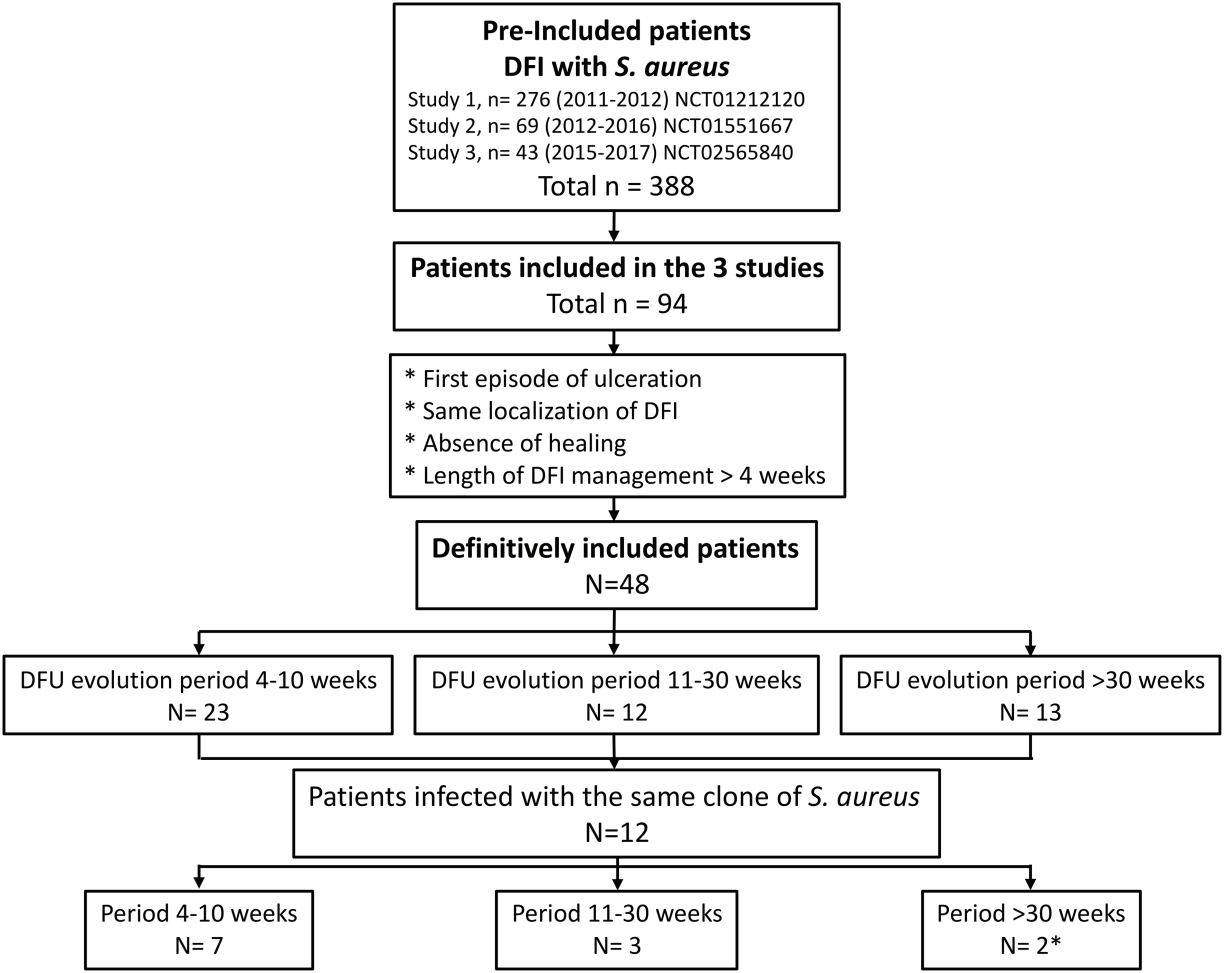
Flowchart of the study. ^*^One of the two patients with the same localization of the diabetic foot infection (DFI) presented two different *Staphylococcus aureus* clones during follow-up.

During a previous 7-year period (2011–2017), we conducted three studies on *S. aureus* isolated during DFI. These studies included 388 patients. Among them, 94 patients participated in the three studies (Figure 1). The patients included in the present study were those with DFI, in the same localization, without healing, and with a DFU management period >4weeks. Thus, we definitively included 48 patients.

Most of the included patients were male (34, 70.8%) with a median age of 69years (27–92) and Type 2 diabetes (44, 91.7%). All DFI were classified as Grades 3 (46, 95.8%) and 4 (2, 4.2%).

During the inclusion period, the majority of patients had two independent tissular biopsies (37, 77.1%), although others had three (9, 18.7%), four (1, 2.1%) or five (1, 2.1%) biopsies.

The follow-up of patients due to non-healing of the wound fell within three timeframes: 23 patients with a follow-up between 4 and 10weeks, 12 patients with a follow-up between 11 and 30weeks, and 13 patients with a follow-up beyond 30weeks.

### Comparative Genetic Diversity of *Staphylococcus aureus* Populations at Inclusion and During Patient Follow-Up

A total of 110 strains were analyzed, 48 isolated at inclusion and 62 isolated during the follow-up period. A great genetic diversity was observed among the 48 *S. aureus* strains isolated from DFI at patient inclusion, as the isolates belonged to 15 distinct genotypes. The 62 *S. aureus* strains isolated during patient follow-up also belonged to 15 genotypes, of which nine had been previously identified. Six genotypes were identified for strains isolated at patient inclusion only and six other genotypes were identified for strains isolated during patient follow-up only (Table 1; Supplementary Table S1).

**Table 1:** Clonal complexes and ST of the *Staphylococcus aureus* isolated from DFI at different periods of patient management.

Genotype (Alere StaphyType DNA microarray)	At inclusion	During follow-up		*p*
	No. of strains and patients (*n*=48)	No. of strains (*n*=62)	No. of patients (*n*=48)^*^	Inclusion vs. follow-up
ST30-MSSA	9 (18.8)	5 (8.1)	5	**0.08**
ST45-MSSA	8 (16.7)	7 (11.3)	6	0.39
CC5-MSSA	6 (12.5)	6 (9.7)	5	0.54
CC8-MRSA-IV	5 (10.4)	9 (14.5)	8	0.78
ST15-MSSA	4 (8.3)	7 (11.3)	7	1
CC8-MSSA	3 (6.3)	5 (8.1)	5	1
ST398-MSSA	3 (6.3)	10 (16.1)	10	0.23
ST72-MSSA	2 (4.2)	0 (0)	0	0.16
CC25-MSSA	2 (4.2)	3 (4.8)	3	1
CC5-MRSA-IV	1 (2.1)	1 (1.6)	1	1
CC9-MSSA	1 (2.1)	0 (0)	0	0.40
CC6-MSSA	1 (2.1)	0 (0)	0	0.40
ST182-MSSA	1 (2.1)	0 (0)	0	0.40
CC59-MRSA	1 (2.1)	0 (0)	0	0.40
ST59-MSSA	0 (0)	2 (3.2)	2	0.50
CC20-MSSA	1 (2.1)	0 (0)	0	0.40
ST22-MSSA	0 (0)	3 (4.8)	2	0.27
CC133-MSSA	0 (0)	1 (1.6)	1	1
ST97-MSSA	0 (0)	1 (1.6)	1	1
CC101-MSSA	0 (0)	1 (1.6)	1	1
CC121-MSSA	0 (0)	1 (1.6)	1	1

MSSA, methicillin-susceptible *Staphylococcus aureus*; MRSA, methicillin-resistant *Staphylococcus aureus*. Statistical analysis was performed using the Fisher’s exact test. Values of *p*<0.01 were considered as significant (in bold).

* A same patient can have DFI with different clones during the follow-up.

At inclusion, ST30 represented the main genotype identified (*n*=9) followed by ST45 (*n*=8) and CC5 (*n*=6). The MRSA clones were rarely identified (7, 14.6%) belonging to the Lyon clone (CC8-MRSA-IV; *n*=5), the CC5-MRSA-IV clone (*n*=1), and the ST59-MRSA-V clone (*n*=1).

During the follow-up of the DFI, ST398 and the Lyon clone were mainly detected (10, 16.1% and 9, 14.5%, respectively), followed by ST45 and ST15 (7, 11.3% both; Table 1; Supplementary Table S1). No statistical difference in the repartition of *S. aureus* clones was noted between the two periods except for the ST30-MSSA clone that was significantly less detected during the follow-up of the DFI. The ST398-MSSA and CC22-MSSA clones were more frequently isolated during follow-up, whereas the ST72-MSSA clone was absent during the follow-up; however, these trends were not significant (*p*=0.23 and 0.27, respectively).

### Longitudinal Evolution of *S. aureus* Clones

**Figure 2:**
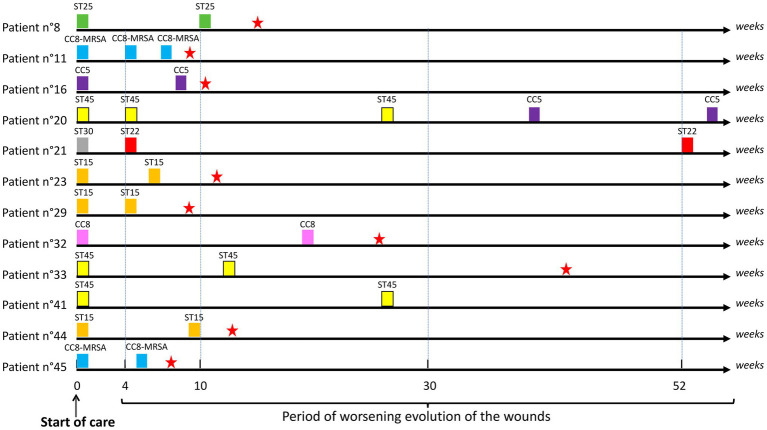
Schematic representation of the periods of identification of identical *Staphylococcus aureus* clones isolated from patients with the DFI. ST, sequence type; CC, clonal complex. Presence of a red star corresponds to the period of wound healing.

During the consecutive 7-year period of three studies performed in our diabetic foot population, about half of the patients (51.1%) had a persistent *S. aureus* colonization/infection of their DFU, harboring one clone (*n*=11, 22.9%), two clones (*n*=28, 58.3%), three clones (*n*=8, 16.7%), or four clones (*n*=1, 2.1%). Among these patients, only 12 (25%) presented a persistent DFU colonization by the same strain (28 strains recovered in patients 8, 11, 16, 20, 21, 23, 29, 32, 33, 41, 44, and 45; Figure 2; Supplementary Table S1). The majority concerned patients with wounds followed over a short period of time (4–10weeks; *n*=7/12; Figure 1). The number of patients with identical *S. aureus* isolated over time significantly decreased along with the increase of the follow-up period (30.4% for the 4–10-week period, 25% for the 11–30-week period, and 15% when follow-up exceeded 30weeks; *p*=0.089; Figures 1, 2). These persistent *S. aureus* belonged to seven clonal complexes (Figure 2). The ST15-MSSA (*n*=3), CC8-MRSA-IV (*n*=2), and CC25-MSSA (*n*=1) clones were only successively detected in the first period of the follow-up. The ST22-MSSA clone was the only genotype still identified after a long period of almost 1-year follow-up (week 4–52 in patient no. 21). Interestingly, this patient had an initial DFU with another *S. aureus* clone (ST30-MSSA) at inclusion that was never isolated again during the patient’s follow-up. Patient no. 20 was the only patient who presented a DFU co-colonized by two MSSA clones, the ST45-MSSA and the CC5-MSSA clones, over long periods of 27 and 23weeks, respectively.

Among the seven patients (14.5%) who harbored MRSA at inclusion, only two continued to maintain a strain of identical genotype over 5–7-week periods (patients 11 and 45, CC8-MRSA-IV; Supplementary Table S1). Among the patients with DFI due to MSSA at inclusion (*n*=41), seven (17%) had an MRSA clone during their follow-up: six with the Lyon clone CC8-MRSA-IV and one with the New Paediatric clone CC5-MRSA-VI. Interestingly, two of these seven patients had other samples during their follow-up and in both cases, the MRSA clone was never detected again and was replaced by the MSSA clone distinct from the initial colonizing MSSA (patients 1 and 46; Supplementary Table S1). We also noted that the median time for harboring MRSA was 4weeks (range 4–24).

A general comparison of virulence gene content detected by the DNA arrays between clonal and non-clonally related groups of *S. aureus* strains (same or different *S. aureus* strains isolated at inclusion and during the follow-up) indicated that *tst* and *agrIII* genes were significantly associated with the group of non-clonally related *S. aureus* (0 vs. 12 and 0 vs. 14, respectively; *p*<0.01; Supplementary Table S2). No other difference was detected.

Among the group of clonally-related strains, only three pairs of isolates (23%) had no difference when comparing the strains at inclusion and during the follow-up (Table 2). The majority of strains had differences in resistance genes content (9/13 pairs or trios of strains), whereas the main virulence genes were less divergent (6/13 pairs or trios of strains). Concerning resistance genes, strains isolated during the follow-up of six patients had lost genes (6/13, concerning C1BP158, C02P12, C02P14, C01P65, C01P78, and B71 isolates). However, the group of genes *blaR, blaI*, and *blaZ*, which encodes for penicillinase, was the most frequently modified and was mainly acquired by *S. aureus* isolates during the follow-up (three patients; C01P54, C02P12, and C04P08 isolates). Regarding virulence genes, in all the six cases showing modifications, genes were lost during the follow-up. Interestingly, three strains (C1BP147, C02P12, and C01P65) had lost the pair of genes that encodes factors associated with the mediation of immune evasion of *S. aureus* in humans (*sak* and *scn*).

**Table 2:** Differences in the gene content detected by the Alere StaphyType DNA microarray® between *Staphylococcus aureus* isolated over time from diabetic foot ulcers (DFU) in the 12 patients of the study with successively isolated strains of identical genotypes (*n*=28).

Patient	Strain designation	Isolation time	Differences in the array results for the 333 screened genes	
			Resistance genes	Virulence genes
8	C1BP44	Inclusion	*msrA*−	*sak+, scn+*
	C1BP147	Follow-up (10weeks)	*msrA+*	*sak*−, *scn*−
11	C1BP108	Inclusion	*merA+, merB+, qacA+, merR+*	–
	C1BP141	Follow-up (4weeks)	*merA+, merB+, qacA+, merR+*	–
	C1BP158	Follow-up (7weeks)	*merA*−, *merB*−, *qacA*−, *merR*−	–
16	C1BP131	Inclusion	–	–
	C1BP185	Follow-up (8weeks)	–	–
20	C1BP139^¶^	Inclusion	*tetM*−, *blaR*−, *blaI*−, *blaZ*−	*vwb+, lukS+*
	C1BP151^¶^	Follow-up (4weeks)	*tetM+, blaR+, blaI+, blaZ+*	*vwb*−, *lukS+*
	C01P54^¶^	Follow-up (27weeks)	*tetM+, blaR+, blaI+, blaZ+*	*vwb*−, *lukS*−
	C01P86^¶¶^	Follow-up (36weeks)	*tetM+, blaR*−, *blaI*−, *blaZ*−	*scn+, sak+, sea+, sed*+, *sej*+, *ser*+, *xylR*+
	C02P12^¶¶^	Follow-up (59weeks)	*tetM*−, *blaR+, blaI+, blaZ+*	*scn*−, *sak*−, *sea*−, *sed*−, *sej*−, *ser*−, *xylR*−
21	C1BP152	Follow-up (4weeks)	–	–
	C01P19	Follow-up (52weeks)	–	–
23	C1BP175	Inclusion	–	–
	C1BP200	Follow-up (6weeks)	–	–
29	C01P32	Inclusion	*blaR+, blaI+, blaZ+*	*–*
	C02P14	Follow-up (4weeks)	*blaR*−, *blaI*−, *blaZ*−	–
32	C01P43	Inclusion	*qacA+, ermC+*	*splE+, scn+, sak+*
	C01P65	Follow-up (16weeks)	*qacA*−, *ermC*−	*splE*−, *scn*−, *sak*−
33	C01P53	Inclusion	*tetM+*	–
	C01P78	Follow-up (12weeks)	*tetM*−	–
41	C02P16	Inclusion	*blaR*−, *blaI*−, *blaZ*−	*sel+, sec+*
	C04P08	Follow-up (27weeks)	*blaR+, blaI+, blaZ+*	*sel*−, *sec*−
44	B52	Inclusion	–	*sej+*
	B116	Follow-up (9weeks)	–	*sej*−
45	B61	Inclusion	*ermC+, qacC*−	–
	B71	Follow-up (5weeks)	*ermC*−, *qacC+*	–

¶ *Staphylococcus aureus* ST45, clone 1.

¶¶ *Staphylococcus aureus* CC5, clone.

### Genome Sequencing of Early and Late *S. aureus* Isolates From DFU

**Figure 3:**
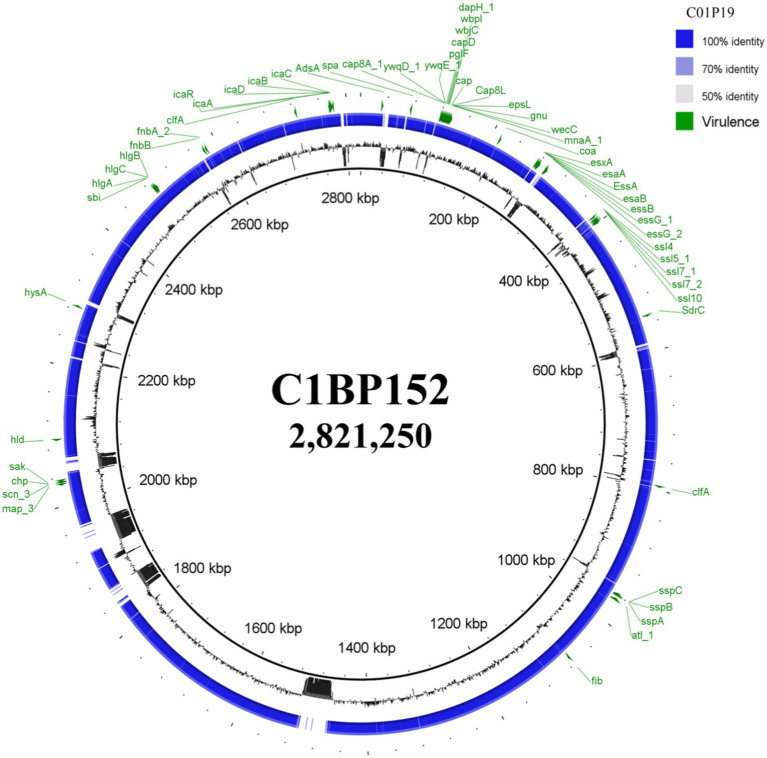
BLAST Ring Image Generator software (BRIG) analysis of *Staphylococcus aureus* genomes isolated from a same diabetic foot ulcer at a 48-week interval. Genome of C01P19 isolate was compared against the genome of C1BP152 isolate and virulence factors obtained from the virulence factor database (VFDB) analysis were annotated on the circular genome of each strain. Circular genome representation was done using BRIG (Alikhan et al., 2011). In green, the main virulence factor-encoding genes known in *Staphylococcus aureus*.

To evaluate the *S. aureus* genome adaptation during DFI, we sequenced the genomes of the two clonally-related strains isolated after the longest period in patient 21 (C1BP152 and C01P19 isolated 48weeks apart in the same wound). The first analysis confirmed that the two isolates were closely related according to Ankrum and Hall (2017) criteria (18), belonging to ST22. The genome sizes were similar: 2,821,250bp for C1BP152 and 2,821,266bp for C01P19 (Table 3). Whole genome comparison obtained by the BRIG (Alikhan et al., 2011) is shown in Figure 3.

**Table 3:** Genomic features of *Staphylococcus aureus* strains isolated 48weeks apart in a patient with diabetic foot infection.

Strain	Date of isolation (DD/MM/YYYY)	Genome coverage (fold)	Sequence type	Genome size (bp)	GC DNA content (%)	No. of open-reading frame (ORFs)
C1BP152	26/06/2012	101×	ST22	2,821,250	33%	2,455
C01P19	28/06/2013	101×	ST22	2,821,266	33%	2,468

Variant call analysis showed the presence of SNPs within coding regions, comparing *S. aureus* isolates at the two time points. When evaluating SNPs in C01P19 strain, 123 divergent nucleotides were identified, and affected 5.8% of the genes (143/2468). Among these differences, 68 SNPs concerned missense and nonsense (stop gain) mutations affecting genes that were classified according to their functions (virulence factor-encoding genes/other genes).

Fourteen stop gain mutations directly affecting both virulence factor-encoding genes and other genes were identified in the genome of strain C01P19 (Table 4). However, none were detected in the main virulence genes known in *S. aureus* (Figure 3). In addition, no mutations were detected in antibiotic resistance genes.

**Table 4:** Genes with stop gain mutations identified by comparison of *Staphylococcus aureus* strains isolated 48weeks apart in DFU of patient 21.

C1BP152 strain/C01P19 strain
Genes encoding for virulence factors
Beta-barrel assembly-enhancing protease BepA Sensor protein SrrB Transcriptional regulatory protein VraR O-acetyltransferase OatA
Genes encoding proteins other than virulence factors
Putative lipoprotein Argininosuccinate synthase Lipoyl synthase Heptaprenylglyceryl phosphate synthase Phosphoribosylaminoimidazole-succinocarboxamide synthase Outer membrane lipoprotein RcsF Demethylrebeccamycin-d-glucose *O*-methyltransferase Chromosome partition protein Smc Poly(glycerol-phosphate) alpha-glucosyltransferase Membrane protein YdfJ

## Discussion

*Staphylococcus aureus* represents the main pathogen isolated in DFI in Occidental countries (Dunyach-Remy et al., 2016). To date, no longitudinal study has evaluated the ability of this species to persist in DFU over long periods of time, yet the knowledge of this persistence is crucial to understand the bacterial adaptation in this specific environment. Among our patients, only 12 (25%) presented a persistent DFU colonization by a related strain during a period ≥4weeks with a median persistence of 12weeks. Only one patient presented successive *S. aureus* strains belonging to the same clonal lineage over time for an extended period exceeding 30weeks (patient 20). These results suggested that long-term persistence of *S. aureus* in DFI after initial colonization is a rare finding in DFI (2.1% of the patients in our study), a low implantation rate compared with other chronic conditions like lung colonization in *CF* patients (Kahl et al., 2003). We assume that the dynamics of *S. aureus* implantation in DFU differ from the dynamics of bacterial colonization and adaptation in the lower respiratory tract during *CF* due to distinct environmental conditions and infection management (Kahl et al., 2003; Vandenesch et al., 2012). Our results support that DFI caused by *S. aureus* is mainly recurrent due to iterative reinfection. This could be explained by the efficiency of the debridement performed in our center by a trained orthopedist and of the antibiotics. This observation is reinforced by the high diversity of *S. aureus* clonal lineages identified in DFI at inclusion and during the follow-up.

Among *S. aureus* clonal lineages in the chronic wounds of diabetic patients, MRSA was identified in 14.5% of the patients at inclusion. This observation could be explained by the inclusion criteria because only patients with a first episode of the DFU were included in our studies. MRSA persisted in wounds for short periods of time only (mean of 4weeks) and was mainly replaced by MSSA clones. More patients (9, 18.8%) had MRSA during their follow-up and one had the same CC8-MRSA-IV Lyon clone identified in three specimens sampled over a 7-week period (patient 11). This patient had a Grade 4 DFI and was hospitalized for 3weeks, possibly explaining this persistence. However, due to both their low representation and implantation rate in DFU, the potential of virulence of these MRSA strains remains contentious. Similarly, the fact that MRSA could be less adaptable to the DFU environment and/or more “sensitive” to the debridement due to a more superficial localization in the wound remains on open hypothesis.

Interestingly, the comparison of the distribution of virulence genes between clonal/persistent and non-clonally-related/sporadic strains isolated at different periods in the same patients showed a significant difference concerning *tst* and *agrIII* genes (Supplementary Table S2). The absence of the *tst* gene but also of other major toxinogenic factor-encoding genes (e.g., *lukF/S-PV, etA, etB, edinA*, and *edinC*) and the low prevalence of *etD* and *edinB* genes in persistent *S. aureus* suggests that these toxinogenic strains do not have the potential to adapt to the DFU environment. They have high virulence and invasiveness and the host immune response is more activated to eliminate these pathogens (Horn and Kielian, 2021). Recently, we also observed, in an *in vitro* model mimicking the DFI environment, that the strains harboring *lukF/S-PV* and *edin* genes were particularly affected by this condition (Pouget et al., 2020). A significant reduction of bacterial virulence can be observed, thus, the immune system could more easily eliminate the toxinogenic strains.

Comparative gene contents of *S. aureus* isolates obtained sequentially over several weeks revealed that the majority of strains (58.3%) showed no modification over time, but among the virulence factor-encoding genes that were lost in persistent isolates, two factors associated with mediation of immune evasion of *S. aureus* in humans (*scn* and *sak*) were lost by three strains. These factors can specifically modulate the human innate immune system, and are considered as mechanisms of *S. aureus* adaptation to the human host (Senghore et al., 2016). Interestingly, the median time to the loss of virulence genes was high [21.5weeks (range 9–52)]. In DFI, the intrahost adaptation is a continual process, where the decrease of virulence could be a key point to limit the host immune response and favor the chronicity of the lesions, as previously suggested (Pouget et al., 2020).

Concerning the resistant gene contents, we observed a similar evolution with four and three isolates having either loss or gain of resistance-encoding genes, respectively, and two isolates having both gain and loss of these genes (Table 2). In the cases of gene loss, *qacA, tetM*, and *ermC* were the most prevalent. These genes encode for resistance to antiseptics, tetracyclin, and macrolides, respectively. They are associated on mobile genetic elements and are easily acquired or lost by bacteria (Anitha et al., 2016). In the five patients (no. 11, 20, 32, 33, and 45) harboring these *S. aureus* showing evolution during DFI, none of the antibiotics or antiseptics used could explain the loss of these genes (data collected in the three previous studies and cross-referenced with the results of this study). In the cases of gene gain, the acquisition of β-lactamase-encoding genes was the most commonly observed. The prescription of amoxicillin/clavulanic acid during the management of these ulcers in patient 20 and 41 could have favored the resistance selection. Although, the *blaZ* gene was particularly frequent in our population and *qacA* present regardless of the period of the isolation of the *S. aureus* strains, *tetM* and *ermC* were rarely identified, but in all cases these genes were present in strains isolated at inclusion and lost in the follow-up strains, suggesting that they could be more susceptible than others to be lost during the adaptation of *S. aureus* to the DFU environment.

To gain insight into the adaptive ability of *S. aureus* in DFU, we compared the genomes of clonal *S. aureus* isolated from the same wound after 48weeks. No modification in the genome size was observed, suggesting that the bacterial adaptation in this hostile environment does not need the reduction of the genome, as previously observed for *Escherichia coli* (Lienard et al., 2021). However, some genome modifications were noted concerning important virulence factor-encoding genes. Indeed, four genes were affected by a stop codon affecting two main bacterial functions: the bacterial defense (*bepA* and *oatA*) and the two-component systems (TCS) SsrA-SsrB and VraS/VraR (*ssrB* and *vraR*). Beta-barrel assembly-enhancing protease (BepA) acts both as a chaperone and a metalloprotease. BepA maintains the integrity of the outer membrane both by promoting assembly of the outer membrane proteins (OMPs) and by degrading misfolded OMPs (Lütticke et al., 2012; Narita et al., 2013; Shahrizal et al., 2019). *O*-acetyltransferase (OatA) is an *O*-acetylating enzyme present in Gram-positive bacteria (Jones et al., 2020). *O*-acetylation of the peptidoglycan protects bacteria from the lytic activity of lysozyme, an enzyme integral for one pathway of innate immune response inside phagocytic cells (Bera et al., 2005). Different bacterial enzymes can modify the essential cell wall polymer peptidoglycan by *O*-acetylation, thus representing an important bacterial virulence factor. Moreover, *S. aureus* possess different members of the TCS, which is involved in the global regulation of multiple targets that determine virulence, stress tolerance, and persistence in these bacteria (Shankar et al., 2015). Among those, the TCS VraS/VraR has been shown to be involved in the control of the cell wall peptidoglycan biosynthesis. This system inhibits the host autophagic flux and delays the early stage of autophagosome formation, thereby promoting bacterial survival. It facilitates the ability of *S. aureus* to resist host polymorphonuclear leukocytes-mediated phagocytosis and killing, thus contributing to immune evasion (Belcheva and Golemi-Kotra, 2008). Mutations in this VraSR system are involved in increasing resistance to antibiotics (Galbusera et al., 2011; McCallum et al., 2011; Qureshi et al., 2014). Finally, SrrA/B is also a member of the TCS, which regulates a type III secretion system and is thus involved in the global regulation of staphylococcal virulence factor production. This TCS regulates different genes involved in bacterial survival under modified environmental conditions such as anaerobic condition, oxidative environment, cytochrome biosynthesis, and assembly, in biofilm formation and programmed cell death. Recently, SrrA/B has been identified to play a role in host-derived nitric oxide resistance (Yarwood et al., 2001; Tiwari et al., 2020). Altogether, these four mutations indicate that during long-term intrahost evolution, *S. aureus* decreased its potential to adapt to the stress environment and its ability to hijack the immune system through virulence attenuation. This could explain the low persistence of *S. aureus* in DFU and a “preference” of the bacteria for an intracellular life (Rasigade et al., 2016). However, we could not exclude the influence of the “environment” encountered by *S. aureus* in this chronic wound. If we previously highlighted that the diabetic foot medium modifies the virulence of *S. aureus* (Pouget et al., 2021), we also know that the competition between microorganisms in these polymicrobial infections directly impacts the invasiveness of *S. aureus* (Pouget et al., 2020). The importance of the bacterial adaptation could also be observed by the formation of small colony variants (Tuchscherr et al., 2020), as previously observed in isolates from *CF* patients (Schwerdt et al., 2018) and DFU (Cervantes-Garcia et al., 2015). All these multifactorial aspects influence the behavior of this pathogen and could explain the modulation of the *S. aureus* virulence. Further work on the transcriptomic profile of these persistent isolates must be developed to evaluate the regulation of the different pathways involved in this long-term evolution.

This study is the first longitudinal one in DFI; it highlights a low prevalence of long-term persisting *S. aureus* infection observed in 2% of the patients. This work provides the basis for the understanding of *S. aureus* colonization dynamics in chronic wounds.

## Data Availability Statement

The datasets presented in this study can be found in online repositories. The names of the repository/repositories and accession number(s) can be found in the article/Supplementary Material.

## Ethics Statement

The studies involving human participants were reviewed and approved by CPP sud Mediterranée III. The patients/participants provided their written informed consent to participate in this study.

## Author Contributions

AS and J-PL: conceptualization, resources, and project administration. MH, CD-R, AY-M, BS, AS, and J-PL: methodology. MH and AY-M: software. J-PL, MH, HM, and AS: validation. J-PL, MH, CD-R, HM, and J-PL: formal analysis. J-PL, MH, CD-R, AB-D, and AS: investigation. AB-D, SS, NC, CD-R, VM, and J-PL: data curation. AS, HM, and J-PL: writing – original draft preparation. MH, CD-R, AB-D, SS, NC, AY-M, VM, and BS: writing – review and editing. HM, AS, CD-R, BS, and J-PL: visualization. AS and J-PL: supervision. AS, BS, and J-PL: funding acquisition. All authors contributed to the article and approved the submitted version.

## Conflict of Interest

The authors declare that the research was conducted in the absence of any commercial or financial relationships that could be construed as a potential conflict of interest.

## Publisher’s Note

All claims expressed in this article are solely those of the authors and do not necessarily represent those of their affiliated organizations, or those of the publisher, the editors and the reviewers. Any product that may be evaluated in this article, or claim that may be made by its manufacturer, is not guaranteed or endorsed by the publisher.

## Footnotes

^1^ www.eucast.org

^2^ http://www.mgc.ac.cn/VFs/main.htm
