# Supplementary material for: Long-Term Intrahost Evolution of Staphylococcus aureus Among Diabetic Patients With Foot Infections
Source: Front Microbiol. 2021 Sep 6;12:741406. doi: 10.3389/fmicb.2021.741406 (PMC8452158; doi:10.3389/fmicb.2021.741406)
Supplement: Supplementary file 2 [file Table_2.pdf]

Table S2. Distribution of the main virulence factor-encoding genes between clonal and non-clonally-related *S. aureus* strains isolated at patient inclusion and follow-up.

	**Clonal *S. aureus* isolated at inclusion and during the follow-up** (n=28 strains) (%)	**Non-clonal *S. aureus* isolated at inclusion and during the follow-up** (n=82 strains) (%)	***P*-value**
sea	6 (21.4)	28 (34.1)	NS
seb	2 (7.1)	3 (3.7)	NS
sec	6 (21.4)	9 (11.0)	NS
sed	5 (17.9)	8 (9.8)	NS
seg	15 (53.6)	42 (51.2)	NS
lukF/S-PV	0 (0)	0 (0)	NS
lukD/E	19 (67.9)	41 (50.0)	NS
hla	28 (100)	82 (100)	NS
hlb	15 (53.6)	56 (68.3)	NS
sak	17 (60.7)	53 (64.6)	NS
chp	19 (67.9)	58 (70.7)	NS
scn	23 (82.1)	73 (89.0)	NS
tst	0 (0)	12 (14.6)	0.034
etA	0 (0)	1 (1.2)	NS
etB	0 (0)	0 (0)	NS
etD	2 (7.1)	3 (3.7)	NS
edinA	0 (0)	0 (0)	NS
edinB	2 (7.1)	3 (3.7)	NS
edinC	0 (0)	0 (0)	NS
splA/B	19 (67.9)	42 (51.2)	NS
agrI	18 (64.3)	49 (59.8))	NS
agrII	10 (35.7)	17 (20.7)	NS
agrIII	0 (0)	14 (17.1)	0.019
agrIV	2 (7.1)	2 (2.4)	NS
cap5	15 (53.6)	49 (59.8)	NS
cap8	13 (46.4)	33 (40.2)	NS
bbp	23 (82.1)	69 (84.1)	NS
clfA/B	28 (100)	82 (100)	NS
fnbpA/B	28 (100)	82 (100)	NS

Statistical analysis was performed using the Fisher's exact test. *P*-values <0.01 were considered as significant. NS, not significant.
